# “Dying alone and being eaten”: dog scavenging on the remains of an elderly animal hoarder-a case report

**DOI:** 10.3389/fvets.2023.1161935

**Published:** 2023-08-29

**Authors:** Louise Bach Kmetiuk, Paulo César Maiorka, Alan M. Beck, Alexander Welker Biondo

**Affiliations:** ^1^Department of Veterinary Medicine, Federal University of Paraná, Curitiba, PR, Brazil; ^2^Department of Pathology, Faculty of Veterinary Medicine and Animal Sciences, University of São Paulo, São Paulo, SP, Brazil; ^3^Center for the Human-Animal Bond, Purdue University, West Lafayette, IN, United States; ^4^Department of Comparative Pathobiology, College of Veterinary Medicine, Purdue University, West Lafayette, IN, United States

**Keywords:** animal hoarding, fresh human-meat feeding, forensic veterinary medicine, hoarding behavior, One Health

## Abstract

Animal hoarding and human consumption by dogs have been important but often understudied aspects of the human-animal bond that can be addressed within a One Health framework. No scientific report has focused on dog scavenging on animal hoarders to date, despite isolated reports of dog scavenging on human remains, mostly due to starvation. The phenomenon has been approached as a confounding factor for human forensics. In 2014, the Animal Protection Department of Curitiba City was called to rescue and handle ten small dogs which had scavenged for a week on the human remains of their elderly owner, a potential animal hoarder. At inspection, three dead dogs in early putrefaction were also found in the household. Human autopsy revealed body putrefaction and lack of soft organs. Along with the dental arch, DNA testing was performed on the remains for official deceased identification. Due to the potential public health risks of aggression toward frail human beings and for the control of zoonotic diseases, all ten dogs were euthanized. Subsequent investigations by the crime scene police, homicide police, and autopsy services were unable to establish or rule out natural death, criminal or suicidal poisoning, zoonotic disease (rabies), fatal dog attack, or fatal accidental trauma. A general protocol has been proposed for future approaches to dog scavenging and suspicious killing of animal hoarders, as well an assessment for the potential adoption or euthanasia of animals owned by hoarders in these circumstances.

## 1. Introduction

The phenomenon of dog scavenging is a potential result of animal hoarding. Animal hoarding disorder is a condition characterized by persistent difficulties discarding possessions and animals, leading to an unhealthy living environment and functional impairment. The disorder was first described as multiple animal ownership (1) and later considered an under-recognized issue in a hard-to-access population, requiring the intervention of public health authorities (2). Recognized as a human mental disorder in 2013 and 2022 (3), there have been around 3, 000 cases of animal hoarding in the USA alone, with high recidivism rates and no validated therapy available (4, 5). The impact of hoarding disorder is worsened by the age of individuals, with older adults being more likely to exhibit hoarding behavior, and often affecting women living alone with poor self-hygiene (1). This creates conditions for accidents, diseases, and health risks, as well as potential dog attacks and consumption of human remains. High treatment abandonment rates and unsatisfactory outcomes have been reported for hoarding individuals, even when compared with other non-hoarding obsessive-compulsive disorder individuals (5).

Older individuals (55–94 years of age) were about three times more likely to present hoarding behavior than younger adults (33–44 years). The impact of complications due to the individuals age would worsen the already existing mental problems leading to degenerating human, animal, and environmental conditions (6). Animal hoarders are also more likely to be women living alone (63.8%) with low self-hygiene (65.2%) (7). Such a scenario creates the optimum conditions for disasters, including accidents such as tripping, falling, and fire; disability, diseases, and health risks due to poor sanitary conditions; and fatal outcomes in severe situations (8). Although reclusiveness and the unattendance of unhealthy elderly persons may result in them dying alone, hoarding and human consumption by dogs are understudied aspects of the human-animal bond and One Health approach, which considers multifactorial factors involving humans, animals, and the environment.

On the animal side of One Health, dog attacks involving predation and consumption of human flesh (9) and postmortem scavenging by dogs not linked to dog hunger have been rare but long described. Besides hunger, displacement behavior motivated by confusion and fear may lead to owner post-mortem mutilation by their dogs (10). A study conducted over 25 years also indicated that social hierarchy may be another reason for the attacks, as dogs may challenge owner hierarchical position after death (10). Such a “pack leader” theory of domestic dogs consuming dead owners may be controversial to modern-day thinking, requiring a level of cognitive capacity and theory of mind that science has not demonstrated in domestic dogs to date, highlighting the importance of the case report herein in providing updated information (11). Nonetheless, a disturbingly higher than usual number of human deaths by dog attacks was reported in 2022 in the USA, highlighting that unhealthy human-dog interactions worsened during the COVID-19 pandemic, probably associated with inadequate breeding and rearing and inappropriate socialization (12). Not surprisingly, a recent study by our research group in Curitiba, Brazil has also shown that dog bites should also be approached from a One Health perspective, as such accidents involve human, animal, and environmental associated risk factors (13).

Dog scavenging of people with hoarding disorder has received media coverage. Several media reports can be found related to lone animal hoarders with post-mortem scavenging by dogs, including an old man eaten by his fifty dogs in Ohio, USA (14) and an old woman partially consumed by her forty-six dogs after death of Hepatitis C in Arkansas, USA (15). Police had shot nine due to aggressiveness, and animal control euthanized 27 to prevent Hepatitis C; the last 10 dogs were put down after a veterinarian was attacked (15). Another old woman in Detroit, USA in 2019 (16), and a woman in an advanced decomposition state in São Paulo, Brazil, along with 20 hungry and two dead dogs, were suspiciously killed by starvation or poisoned by ingesting corpse parts (17).

While dog scavenging on human remains has been rarely reported (18), it has been observed in cases of animal hoarders. This case report provides a detailed account of a potential animal hoarder's death followed by dog scavenging, highlighting the public health, animal welfare, and legal issues associated with such incidents.

This case report was part of a study approved by the Ethics Committee in Human Health of the Brazilian Ministry of Health (protocol 3, 166, 749/2019) and the Animal Use Ethics Committee (protocol 077/2015) of the Federal University of Paraná, Brazil. In this particular case, as the deceased had no known guardian at the time and DNA failed to confirm potential relatives, individual consent was not possible. Dr. Biondo himself was the director of the Curitiba City Animal Welfare and Control at the time, ensuring legal responsibility and an official request for post-mortem information and images. In addition, authors waited almost a10-year period and removed all personal information besides age, gender, year, and city of events. Despite the apparent disturbing data and graphic images provided here, no disrespect was intended. Instead, authors support the awareness importance of this case report, as information may help other cities with dealing with such concomitant and degrading human and animal conditions.

## 2. Case presentation

The deceased individual in this case was an elderly person with no known guardian, and no potential relatives could be confirmed through DNA testing. The report focuses on the events and does not disclose personal information.

In June 2014, the Animal Protection Department of Curitiba City was called to rescue a group of small dogs that had scavenged on their owner's remains for a week. The owner lived in a household with significant garbage accumulation and neglected dogs. During the inspection, the house was found to be unkempt and unclean, with feces, garbage, and clutter throughout. Ten dogs were found hidden under the house, and three dogs were found dead. The living dogs exhibited fearful behavior and were given care and temporary shelter.

**Figure 1:**
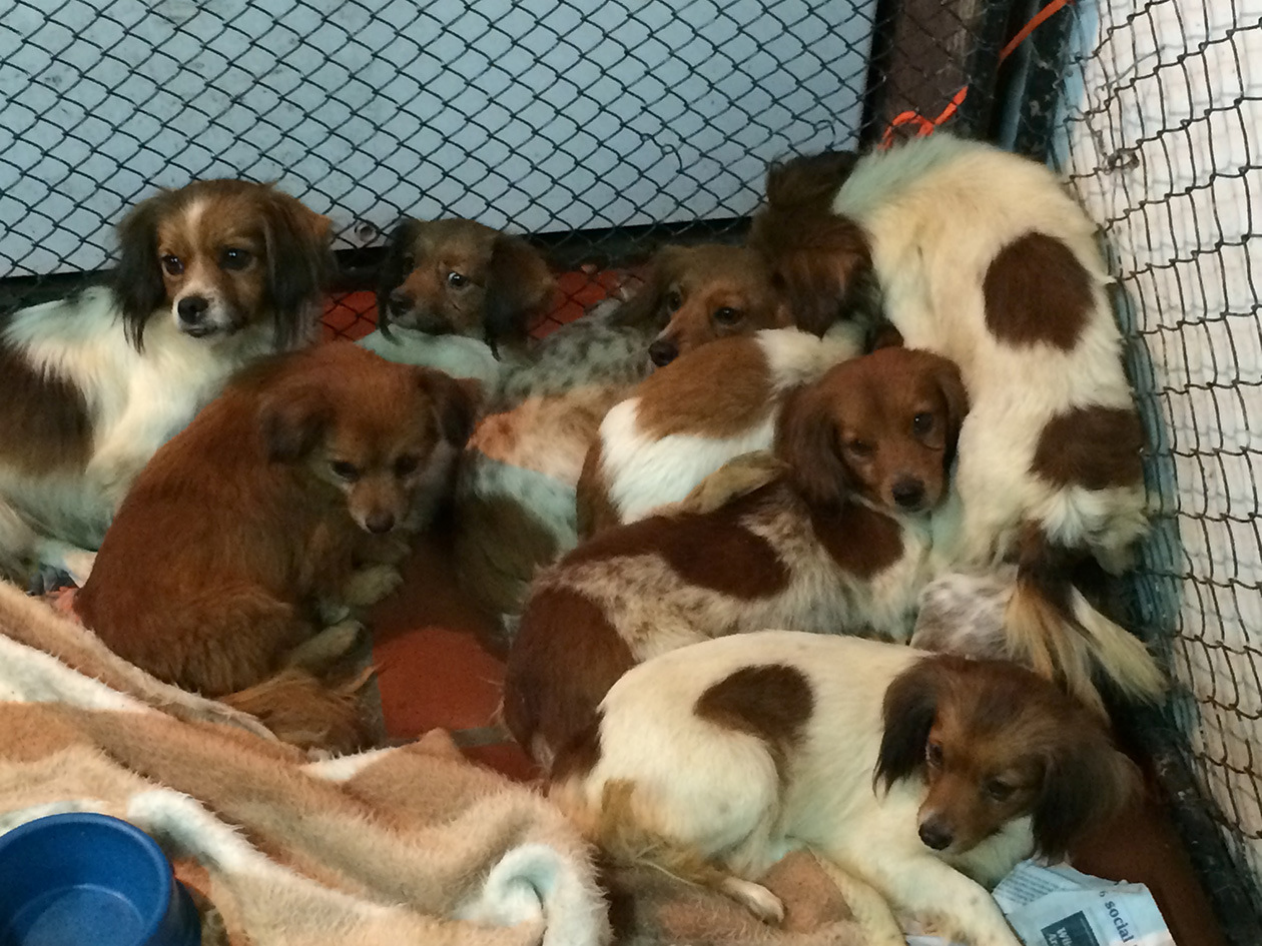
Dogs found at the household.

At the inspection, the house was apparently unkempt, uncleaned outside and inside. Feces, garbage, clothes, dirt, and other clutter objects were found throughout the floor of all rooms. Ten mixed breed dogs were found hidden under the house, and three similar dogs were found dead with a few days of putrefaction. The living dogs refused human contact, were given fresh water and commercial pet food, and kept for a week at the household for treatment and adoption. All ten dogs were small and alike, probably inbred, having normal body score condition, and sharing fearful behavior toward human beings (Figure 1).

The owner's clinical history included a diagnosis of untreated schizophrenia, isolation from family and neighbors, and family neglect and/or inability to deal with his disorder. The deceased was of a reclusive man in his 80s (more than the average life expectancy in Brazil at the time of 75.2 years), living alone with his dogs, likely with no dog walking or other outdoor activities. Potential contact with rabid animals should not be ruled out.

**Figure 2:**
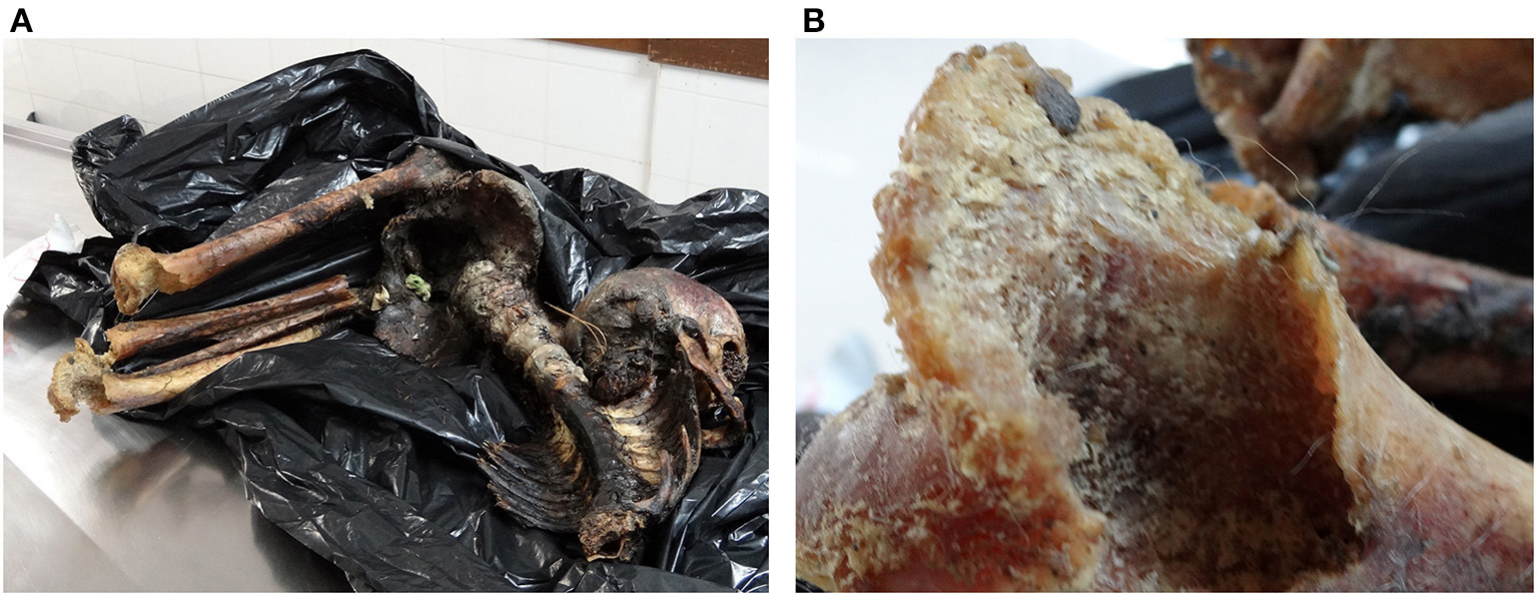
All human remains found at the scene and brought to the forensic medicine facility of Curitiba, Brazil, including a partial backbone, ribs, femur, tibia, and skull **(A)**. Despite consumption and advanced putrefaction, biting marks and dog hair were visibly observed on the femur epiphyses and skull **(B)**.

The human body remains were held at the Curitiba Forensic Pathology Office, which reported at the time the “impossibility of an autopsy” due to “extensive consumption of body parts and advanced putrefaction”. Likewise, no official establishment was made of the date of death, estimated in 2 weeks by history and the presence of a few worms digesting soft tissue on the deceased skull. The skull was intact with no bone trauma, but missing eyes, tongue, and other soft tissue parts, along with both femurs (Figure 2). A single humerus bone was avulsed and aside of the body, found in a different room. The gastrointestinal tract was also partially missing, as well as most muscle tissue on the arms and legs.

As already mentioned, there was a single rabies report within city limits in the past 30 years, which was a cat infected in 2012 by a non-hematophagous bat variant (19). In addition, out of 806 bats collected by the animal control service from citizen complaints from 2010 to 2015, 9/387 (2.3%) tested positive for rabies throughout the city (20). As rabies may change normal behavior, bats may be observed at daylight flying and landing in household yards, which was the reason for most complaints (20). Thus, although the three dead dogs most likely died from starvation, given the only source of food available at the time was the deceased body, rabies was unlikely but could not be ruled out in this case. Dead dogs were also in an advanced stage of putrefaction, and carcasses were immediately discharged without examination of dog gastric contents. Despite no behavioral assessment being made at the time, the ten surviving dogs were considered healthy by inspection and displaying aggressive behavior toward the city veterinarians. Dogs were all adults and alike in size, color, and shape, apparently related and inbreeded. For the ten living dogs, immediate chemical sedation, anesthesia, and humane ethic euthanasia were performed following the Brazilian Veterinarian Association standards. Dogs were subjected to euthanasia due to public health risks, including rabies and aggressive behavior toward children, elderly, and disabled people. Dogs were clinically healthy at the time and false negative results were expected for rabies on brain tissue, even under peripheral infection. In addition, the anti-rabies vaccination status of suspicious dogs was unknown. Finally, no compulsory testing was required, as no suspicious human contact was made besides the deceased. Since human and dog nervous tissue samples were inappropriate for post-mortem rabies diagnosis due to advanced putrefaction, rabies was not eliminated as the cause of death.

Despite efforts, no further information was obtained about the deceased, even from adjacent neighbors, who just confirmed the elderly individuals' reclusiveness, self-isolation, lack of self-hygiene, and presence of multiple dogs. Available police reports lacked such information as well. As no previous complaints were made, no records were found in the city services either, as no specific active service existed at the time. In fact, the city (animal and object) hoarding taskforce responsible for surveying, mapping, monitoring, and prevention was only created in 2015, in response to this and other tragic outcomes, including a woodhouse fire by a candle (unpaid electric power was cut off) where a single elderly woman lived, killing 43 dogs indoors and releasing around 40 dogs on the streets (21). Even after the establishment of the city taskforce, animal hoarding remained a difficult diagnosis, as psychiatric confirmation could take months to years, due to the refusal of patients to attend medical appointments, lack of psychiatrist's agenda to visit patients at their households, and the need of a sequence of appointments to exclude other potential underlying disorders. In addition, the term “hoarder” recently became derogatory and of exclusive psychiatric use. As of June 2023, the Curitiba city actively monitored around 250 people with “hoarding behavior”, a current non-pejorative term to describe the disorder without the need for an established diagnosis.

## 3. Discussion

Hoarding, including animal hoarding, is a worsening mental disorder that negatively affects the affected individuals' self-esteem, hygiene, and self-care. Elderly hoarders are particularly at risk due to their isolated and neglected living conditions (8, 22). In addition to medical conditions, elderly hoarders often have less frequent medical visits (23).

Dogs owned by hoarders are highly stressed and require re-socialization before potential adoption. The behavior of these dogs can pose public health concerns, especially for physically vulnerable individuals. Proper evaluation and screening for zoonotic diseases should be conducted before considering adoption (4). In addition, all adopters should be given full information on the dog's history and sign an awareness letter on potential abnormal behavior. The potential individual and/or “pack behavior” of the ten feral-like dogs relating to future aggression toward physically fragile human beings including children, elderly, and disabled people were considered public health concerns. As a previous study has shown, the stomach contents obtained by feces from dogs at the scene may help identify whether the dog has ingested human tissue (24). Previous studies of postmortem predation and decapitation have shown human parts and belongings such as teeth, bones, fingernails, clothing, earrings, and jewelry in the feces and X-rays (24, 25). Animal scavenging after human poisoning may also result in animal death (26), so the case here raised concerns as the owner and three dogs were found dead and in a state of advanced putrefaction.

Besides behavior evaluation, if adoption was considered, a complete serological and molecular screening should be performed for potential zoonotic diseases including rabies, leptospirosis, and salmonella. However, before that, if rescue was considered, the rescue personal should be aware and the animal shelter or animal service facilities should be prepared for a safe animal quarantine, under careful handling and isolated from the main animal shelter population. Sheltering suspicious rabid dogs could result in a similar situation to the North Dakota Animal Shelter, in which an unknown rabid stray dog housed for 12 days culminated in a massive human rabies postexposure vaccination, and the euthanasia of 25 in-shelter and 11 post-adoption dogs (27).

Current animal cruelty laws often fail to respond adequately to animal hoarding cases, and intervention typically occurs at critical stages of harm (5). As a result, dogs scavenging on human remains may potentially occur in other communities. Crime scene investigations involving animal hoarders should involve veterinarians to address the risk of infectious diseases.

The One Health approach has been essential in addressing hoarding disorder, as it involves the interconnectedness of human, animal, and environmental health. Recognizing, preventing, and responding to animal hoarding requires community-based approaches and increased awareness (5). Such a multidimensional issue with public health, human health, and animal welfare implications has been described in the first animal hoarding reports in Spain, involving 27 cases from 2002 to 2011 (28). Despite multidisciplinary actions and different stakeholder involvement, an animal hoarding case in Italy remained unsolved from 2005 to its report in 2020 (29). Such long-term intervention may be required due to time-consuming (sometimes ineffective) efforts on legal, sanitary, and veterinary aspects associated to hoarding reluctance and recurrence (28).

The One Health approach, facts, and outcomes of the present case report have been provided below in an easy-to-follow format.

### 3.1. Human health

Animal hoarding has been described as a degrading mental disorder mostly affecting elderly, lonely, reclusive, and isolated people with concomitant diseases and less frequent medical visits, constantly at risk of unattended traumas, illnesses, and death, and daily exposed to several confused and starving companion animals. Here, the unattended person has died alone and unnoticed, with his innumerous dogs scavenging on his remains.

### 3.2. Animal health

Dogs owned by animal hoarders vary in numbers from a few to hundreds, mostly stressed, in a highly dense and renewable population, lacking veterinarian attendance and a proper nutritional diet, when not starving, and exposed to vaccine-preventable species-specific (parvovirus and distemper) and zoonotic (leptospirosis and rabies) diseases, as not ruled out in this case. Additionally, dogs scavenging on human remains may present traumas and disruptive behavior disorders, posing adoption concerns due to injury risks to children, elderly, the disabled, and other vulnerable individuals. Finally, this case report has urged for an approach protocol and guidelines for effective evaluation of surviving dogs.

### 3.3. Environmental health

The overall living conditions of animal hoarding are unsanitary, unsafe, and predisposed for accidents. Household impairment and movement restriction due to piles of unserved objects and garbage may lead to tripping, falling, and fire. Moreover, poor indoor and outdoor sanitary conditions may result in health risks and other disorders, particularly water, food, and vector-borne and zoonotic diseases. Finally, as observed here, households may be isolated and occupants unattended, leading to potentially fatal outcomes.

One of the strengths of the present report may be the on-field and real-life clinical decision making, which may at first be considered inhumane and unjustified. As already mentioned, the same tough decision had been made previously, based on the strong public health concerns about rabies. As the rabid dog housed in the North Dakota Animal Shelter, USA, resulted in the euthanasia of 36 dogs, including 11 already adopted, as part of emergency measures (27), dog euthanasia here was considered the last option to avoid contact and risk of human and animal infection, as no full isolation could be guaranteed for the suspicious dogs. Based on this report, Animal Services in endemic rabies areas worldwide may be prepared for prompt responses and safe dog isolation in such cases. Although dog euthanasia may comply with local laws applicable to this type of case in certain remote regions of Brazil, Curitiba is the eighth biggest Brazilian city and has been considered the most sustainable (and pet-friendly) city nationwide for decades, recognized as the most sustainable city several times in Latin America (30). In addition, euthanasia of healthy dogs has been banned statewide in Parana since 2012 (31), as in other countries worldwide, indicating physical and behavioral recovery of animals as a first approach instead euthanasia. Nonetheless, as rabies remains a concern in Curitiba, with rabid non-hematophagous bats found every year within city limits, in association with the aggressive behavior of surviving dogs, immediate euthanasia was indicated as necessary for public health at the time, corroborated by previous cases in the USA (27).

As a nationwide model, a series of Curitiba city laws have provided a legal scaffold against animal violence and cruelty, including the mandatory use of dog muzzles in aggressive breeds (9,394/1999), the removal of dog waste (643/2001), responsible guardianship and microchipping (11,474/2005), and penalties in cases of animal cruelty (13,908/2011). In addition, despite statewide rabies vaccination stopping in 2015 (with the exception of the far-west Paraguayan border), dogs, cats, and bats with sudden death or death by neurological signs are sent for rabies diagnosis at the State Reference Laboratory (LACEN), complying with federal guidance of the Brazilian Ministry of Health. Nonetheless, as the microchip system has not been connected to rabies and other vaccinations, no verification can be made without the individual paper-record or vaccination certificate.

The present study has some limitations. Very little detail was available about the deceased individual, including potentially relevant medical history. Therefore, the diagnostic assessment could not be adequately obtained, according to the case report guidelines (CARE). As no confirmed true relative was found, no information on whether medical records even existed was available at the time. In addition, as already mentioned, no autopsy was performed due to the few bones left, which were in an advanced stage of putrefaction. Even so, this case report may contribute as a warning to public services and authorities, along with relatives, friends, and neighbors, on the importance of continuous monitoring and checking on individuals with hoarding behavior, mostly elderly and those living alone with several dogs in unsanitary conditions. In addition, the study should have highlighted the aspects much more and emphasized the need for an interdisciplinary approach involving various stakeholders to avoid sad and tragic epilogs. However, rather than an attempt to provide established protocols, the present case report has aimed to be a starting point on discussion of its most serious outcomes, warning of different One Health issues of animal hoarding including elderly mental illness and loneliness, animal starvation, cruelty, abandonment, household degradation, and impairment, leading to the unassisted death of people and companion animals.

This case report has shown an approach to rabies as a priority problem, aiming above all to protect human health and avoid a possible, perhaps improbable, spread of rabies. Despite complying with restricted public health laws and perspectives at the time, this way of acting may be certainly legitimate, but it also anthropocentric, not complying with the holistic vision of One Health in which the three elements that make it up (humans, animals, and the environment) should enjoy equal dignity and attention. A protocol of approach of dogs scavenging on deceased persons should include the dog behavior, the time of death, the presence of food and water, and the elapsed time from death to scavenging. Also, the dog body condition and presence of food reserves, ideally with veterinary records on essential care for the dogs prior to the episode, should be obtained. Thus, for a more comprehensive perspective, particular attention should be paid to the role that veterinary doctors play in safeguarding animal welfare, possibly starting with refresher courses aimed at giving the appropriate indications for dealing with these cases of animal abuse in future, with adequate infrastructure to fully rule out rabies. Also important are efforts toward behavior recovery of aggressive (and traumatized) dogs, in attempt to allow safe adoption.

## 4. Conclusion

In conclusion, the report here of dog scavenging on an elderly animal hoarder owner has shown the need for a specific protocol for dogs scavenging deceased persons, including the time of human death, dog behavior, presence of food and water, and the elapsed time from death to scavenging. In addition, animal services in endemic rabies areas should be prepared for prompt responses and safe dog isolation sheltering, particularly in animal hoarding cases. Finally, the case report here may serve as a warning to public services and authorities, along with relatives, friends, and neighbors, on the importance of monitoring hoarding behavior individuals, mostly elderly and those living alone with several dogs under unsanitary conditions. Despite no case being on our survey, cats may pose a similar risk.

## Data availability statement

The original contributions presented in the study are included in the article/supplementary material, further inquiries can be directed to the corresponding author.

## Ethics statement

The studies involving human participants were reviewed and approved by the Ethics Committee in Human Health of the Brazilian Ministry of Health (protocol 3, 166, 749/2019), Brazil. The Ethics Committee waived the requirement of written informed consent for participation. The animal study was reviewed and approved by Animal Use Ethics Committee (protocol 077/2015) of Federal University of Paraná, Brazil.

## Author contributions

AMB and AWB conceptualized and designed the study. LK and AWB drafted the initial manuscript and revised the manuscript. AWB designed the data collection instruments, coordinated, and supervised data collection. PM and AMB reviewed and critically revised the manuscript. All authors approved the final manuscript as submitted and agree to be accountable for all aspects of the work.
